# From “learning from the field” to jointly driving change

**DOI:** 10.1057/s41271-021-00280-3

**Published:** 2021-04-27

**Authors:** Joshua Galjour, Thomas Schwarz, Itai Rusike, Marta Lomazzi, Laura Hoemeke, Helen Prytherch, Teurai Rwafa-Ponela, Margaret Nanyonga, Ravi M. Ram, Sunisha Neupane, Peter Tsasis, Maryam Bibi Rumaney, Timothy Akinmurele, Michael Ssemakula, Rituu B. Nanda, Emmanuel Kabengele Mpinga

**Affiliations:** 1 Institute of Global Health, Faculty of Medicine, University of Geneva, 9 Chemin des Mines, 1202 Geneva, Switzerland; 2 The Global Fund To Fight AIDS, Tuberculosis and Malaria, Geneva, Switzerland; 3 Medicus Mundi International Network, Basel, Switzerland; 4 Community Working Group On Health, Harare, Zimbabwe; 5 World Federation of Public Health Association, Geneva, Switzerland; 6 Gillings School of Global Public Health, University of North Carolina, Chapel Hill, NC USA; 7 Swiss Tropical and Public Health Institute, University of Basel, Basel, Switzerland; 8 SAMRC/Centre for Health Economics and Decision Science – PRICELESS SA, University of the Witwatersrand, Johannesburg, South Africa; 9 Kampala, Uganda; 10 People’s Health Movement Africa, Nairobi, Kenya; 11 University of Montreal, Montreal, Canada; 12 School of Health Policy and Management and School of Administrative Studies, York University, Toronto, Canada; 13 Cape Town, South Africa; 14 Equitable Health Access Initiative, Lagos, Nigeria; 15 Human Rights Research Documentation Center and People’s Health Movement, Kampala, Uganda; 16 Global Fund for Children and Institute of Social Studies Trust, New Delhi, India

**Keywords:** Civil society, Primary health care, Health policy, Social accountability, Participatory research, Field

## Abstract

The theme of the 8th edition of the Geneva Health Forum (GHF) was *Improving access to health: learning from the field*. While ‘the field’ often denotes people, patients, communities, and healthcare workers, we challenge the notion and its usage. A group of like-minded conference participants set up a working group to examine the term ‘the field’ and look at questions related to language, power, participation, and rights. By highlighting deficiencies of existing terms and jargon, we explain why language is a form of power that matters in public health. We describe global, regional, and national case studies that facilitate full participation to achieve more equitable health outcomes. By concluding with concrete recommendations, we hope to contribute to these shared goals: to correct power imbalances between health authorities and the people that they intend, and are expected, to serve. The authors are all members of the working group.

## Introduction

The Geneva Health Forum (GHF), a bilateral event bringing together diverse stakeholders in global health, describes itself as “the forum of innovative practices in Global Health” and “one of the most important international conferences on Global Health” [1]. The theme of the 2020 edition, postponed from March to November 2020 and held virtually due to the COVID-19 pandemic, was “*Improving access to health: learning from the field*.” While ‘the field’ may denote people, patients, communities, healthcare workers, and others, we challenge the notion of ‘the field.’ The term limits those ‘in the field’ to research objects to be studied, as those requiring assistance, or as passive beneficiaries of services and policies. “Learning from the field” implies that learning or knowledge and related action and change remain with an ‘external’ or ‘superior’ actor (health authority, service provider, aid agency, research institution, policymakers, or the like). At its extreme, in health research and international development cooperation (‘aid’), people use the term ‘field’ to objectify others, reflecting unequal distribution of power and resources. This inequality persists. In health research, it leaves members of researched communities out of study design and implementation, or retains ownership of data and resultant publications in higher-income countries of the researchers, not those of study participants.

Resolving power imbalances will require efficient strategies to address root causes and manifestations. In our experience, the term ‘in the field’ most commonly refers to low- and middle-income countries (LMICs) as targets of public health interventions or subjects of research led by outside groups. Here, power differentials between researcher and study subjects are often greatest. For this reason, and due to the limits and focus of our analysis, we restrict our analysis of ‘in the field’ to LMICs, rather than to high-income countries.

To examine power implications of the term ‘in the field,’ this Viewpoint focuses on deficiencies in communication in public health policymaking, research, and implementation of public health interventions. It involves case studies of stakeholder groups working towards alternatives. We conclude with policy recommendations for health research and funding institutions to improve collaboration ‘in the field.’

## Objectives

A multisectoral group of representatives from civil society, academia, and funding organizations organized a workshop titled “From ‘learning from the field’ to addressing power imbalances and jointly driving change.” We aim to provide an alternative voice at the GHF, raising questions about power, rights, and ownership of the processes, management, and dissemination of public health research and policymaking. First we highlight the deficiencies in the relationship between health authorities, donors, service providers, research institutions, and the people that they intend, and are expected, to serve. We examine language and its use to explore people’s participation and power in health policymaking and research. Next we review global, regional, and national level case studies provided by working group members that may contribute to overcoming power imbalances and achieving a new agenda for ‘people’s participation.’ Our ultimate aim is to prompt reflection among participants in the GHF and to help the conference evolve from an approach of *learning from the field* to one focused on *jointly driving change*. That is, to move from taking lessons back from ‘from the field’ to actively promoting equity-affirming public health advocacy, research, policy, and practice.

## Background

### Who is learning from whom? A consultative process to examine the conference theme

From September 2019 to January 2020, our group followed appreciative inquiry technique to explore our own understanding and perspectives on “learning from the field to jointly driving change.” Appreciative inquiry can be used as a tool to “support discovery, dreaming, design, and creation of a vision that inspires people…to move to a collective destiny” [2]. We conducted an appreciative inquiry analysis to identify best practices for equitable health research and policymaking using several methods:

- Literature review and analysis of key concepts, including communities, empowerment, ownership, political participation, social accountability, and community engagement (This is not an exhaustive list and relates only to the main topics under discussion).
- Introspective observation by sharing our personal experiences in the field of public health. We drew on previous analytical models, including the work of Xue et al., and Olgunick on the introspective observation method [3, 4].
- Group discussions in the form of coordinated virtual exchanges between experts around the world on the themes noted above.

We identified case studies at national, regional, and global levels through our literature review and complemented it with our personal experience and involvement in many of them as researchers, advocates, and funders of public health interventions. Although far less than a comprehensive list of the universe of work in this area, we believe the initiatives we describe provide a broad overview of some of the most salient examples of ongoing efforts to advance equity in global health research, funding, and practice. Our group held five virtual consensus meetings from October 2019 to February 2020 to debate and arrive at the views expressed here. Debates during our meetings allowed us to combine our thinking and draft this Viewpoint using an iterative process and sharing opinions to reach consensus.

### Language and power matter: of people and participation

In 2018, primary health care (PHC) attracted renewed attention on the fortieth anniversary of the Alma-Ata Declaration. WHO defines PHC as a “whole-of-society approach to health and well-being centered on the needs and preferences of individuals, families, and communities. It addresses the broader determinants of health and focuses on the comprehensive and interrelated aspects of physical, mental, and social health and well-being” [5]. The anniversary re-positioned actions for health with people and communities: “The people have the right and duty to participate individually and collectively in the planning and implementation of their health care” [6]. Such repositioning, however, is too often easy to stipulate among policymakers at global level but difficult for people and communities to put into local practice. Lacking is specificity about the types of reforms and strategies needed to institute the proposed changes, leading to failures to address the structural social and political determinants of health [7, 8] and health policies, sometimes amidst opposing national policies, guidelines, and budgetary allocations.

We see the first challenge as a rhetorical one: inaccurate terms lacking shared meaning inundate the global health vocabulary, littering the field with jargon and hollow buzzwords about people, participation, and power. Observed communication gaps could be a symptom, not the cause, of a larger problem: a power imbalance. ‘Poor wording’ could be a manifestation of broken or unequal relationships resulting from power imbalances. Language matters; acknowledging its power is a key step for building people-centered health research and systems for more equitable outcomes.

For example, ‘citizens’ in different contexts may be individuals or organized groups. With the rise of displacement, migration, and the increasing challenge of providing health for all, despite global commitments for universal health coverage (UHC), the term ‘citizens’ is not always clear. Galjour and Russell have defined ‘civil society’ as a “diverse and complex sector, comprising non-state actors from an array of interest groups, networks, organizations, and institutions” that often share goals [9]. The expressions ‘strengthening civil society’ and ‘defending civil society space’ support the call for people to organize themselves to claim their rights. The term ‘civil society’ requires further definition.

Regional and national authorities commonly specify public health programs and policies, yet public health services are typically implemented at a local level. We adopt the definition of “public health” as put forward by the Editors of this journal that refers to “all activities that affect the health of the population: the environments where people carry on their personal and work lives and that produce the food, air, water, transportation systems, and services on which they depend; nutrition, climate, education, income, race, and poverty, and so on” [10]. Logically then, in such an expansive definition of public health, the ‘community context’ becomes a fundamental determinant of health outcomes. James et al. define ‘community’ as a small social unit, “group or network of persons who are connected (objectively) to each other by relatively durable social relations that extend beyond immediate genealogical ties and who mutually define that relationship (subjectively) as important to their social identity and social practice” [11]. This definition contrasts with an often-simplistic idea of ‘community,’ particularly in international cooperation, where ‘communities’ typically mean small geographical units (often villages), as a core entity for people’s self-organization. Although there are exceptions, researchers and policymakers and other public health professionals use the term ‘communities’ in a monolithic way, ignoring diverse social realities and variations among national health systems and policies. According to the WHO definition, a health system would “include all the activities whose primary purpose is to promote, restore, or maintain health” [12]. We use the term ‘community’ with caution [11], and mostly in the broader sense, and we use the term ‘people’ (as individuals and collectives) in place of ‘citizens.’ We refer to ‘communities’ because terms like ‘community empowerment’ and ‘community participation’ have gained a meaning meriting further reflection.

### From empowerment to participation

‘Empowerment’ is a buzzword in health promotion and health cooperation. ‘Community empowerment,’ as defined by the World Health Organization (WHO), “refers to the process of enabling communities to increase control over their lives” [13]. But if empowerment is understood as being ‘enabled,’ or ‘given’ to the people (in particular to ‘communities’) by external actors, the same question arises as with ‘the field’ discussed above: How can power be ‘given’ to the people, if not claimed and exercised by people themselves?

‘Power’ does not refer to the expression or enjoyment of rights. We need to broaden our understanding of power in its varied dimensions, such as psychological, cultural, social, political, and economic—by not limiting the definition of power to rights, or their expression, alone. Molm summarizes decades of research on power balances (and imbalances), defining power as a “structural potential created by the mutual control that persons exercise over each other’s outcomes.” Molm differentiates between power and its use [14]. Our objective is to explore the expression of power as manifested *through* the enjoyment of individual or collective rights and responsibilities [15].

‘Ownership’ in health policies and health promotion is based on its common-sense definition “as the act, state, or right of possessing something” [16]. It includes ownership of one’s health, or control over one’s life; Nigel Crisp proposes the term “health citizenship” [17]. It also includes public (or not) ownership of the health system, or a democratic (or not) process of ownership in design and implementation of health policies (as in the Alma-Ata Declaration). All these aspects of ownership play important roles in determining the scope of real ‘people’s participation’ in health.

Political participation “includes a broad range of activities through which people develop and express their opinions on the world and how it is governed and try to take part in and shape the decisions that affect their lives” [18]. To fully participate, and to lead social development, people—individuals and groups—must have the power to do so. But can people overcome the power imbalance that prevents them from fully participating as agents or drivers of change? The question is a political one; it cannot be answered “technically” nor just by changing terminology. Language ultimately influences the power that people can exercise over other people.

### People’s participation in design and implementation of health policies

We return to our first objective, to explore language and its use in people’s participation and power in health policymaking. Policies influence local implementation of research findings. These policies then influence practice. The presence of empowered and engaged people with roles in designing and implementing health policies is relatively new in many countries. Expressions of this are as diverse as the states and interpretations and instruments of democracy. These range from a high level of formalization and institutionalization at national and subnational levels to realities of oppression or poor governance where participation has little space and must be expanded through resistance and social action. In claiming their rights, people’s movements often call for policy change at national and even international levels. People’s participation and engagement in planning, decision-making, and implementing policies lead to empowerment and redistribution of power, but also to increased knowledge, authority, and problem solving [19, 20].

### From decentralization to social accountability

Decentralization of national health systems moves decision-making away from centralized control and closer to the users of health services. The World Health Organization (WHO) has promoted this for decades to overcome the ineffectiveness of national health administrations [21]. WHO’s definition of decentralization includes:

“…political decentralization…” to “…give citizens or their elected representatives more power in public decision-making. Its goal is to introduce more participatory forms of governance by giving citizens, or their representatives, more influence in the formulation and implementation of health policies and plans” [21].

Malena et al. define social accountability as “an approach toward building accountability that relies on civic engagement, i.e., in which it is ordinary citizens and/or civil society organizations who participate directly or indirectly in exacting accountability.” [22]. We appreciate that this definition focuses on “citizen-driven accountability measures” that strengthen links between an informed and active people’s participation and public officials [22]. This model most closely resembles the types of measures and initiatives on which we work.

Indeed, proliferation of movements, initiatives, guidelines, tools, and methodological advances from 2010 to 2020 illustrate growing interest in social accountability. These movements for participation and social accountability directly question power imbalances and demand (re)distribution of power. At the time of writing this paper, the Partnership for Universal Health Coverage (UHC2030) had been working closely with the WHO to draft a “Handbook on Social Participation for UHC,” launched in December 2020 [23]. According to WHO,

UHC means that all individuals and communities receive the health services they need without suffering financial hardship. It includes the full spectrum of essential, quality health services, from health promotion to prevention, treatment, rehabilitation, and palliative care [24].

The objective of this Handbook is to assist WHO Members States increase social participation and community engagement in the health sector, acknowledging that social participation requires skills in which governments will have to invest further to reach UHC goals [23].

### Technical and normative guidance on community engagement

‘Community engagement’ is an important concept in achieving the PHC vision enshrined in the Alma-Ata Declaration that stretches back more than forty years. The WHO Member States clarified in 2016 that “empowering and engaging people and communities” is the first strategic approach in the WHO Framework on Integrated People-Centered Health Services [25]. The WHO positions principles of “community” and “community engagement” as key pillars in attainment of UHC goals, as health systems worldwide often are over-stretched to respond to the 21st Century’s “global public health” issues that require interdisciplinary responses [26].

### The role of aid in deepening or overcoming power imbalances

In addressing power imbalances in the design and implementation of health policies, the role of international cooperation (‘aid’) and its actors deserve attention. While certain patient groups and ‘communities’ in low- and middle-income countries might benefit from attention and support given to them by the aid sector, the key question to be answered is the same as for other aspects of ‘aid.’ This question is part of a critical analysis of the “relevance, legitimacy, and effectiveness”—in a debate that is as old as aid itself [27].

- Does aid (particularly when it situates people and ‘communities’ at its core) allow people to better claim their rights and properly engage in changing the root causes of their realities?
- Alternatively, do aid and its actors distort people-centered national health policies, systems, and negotiation processes?

Voices of those most affected by health inequity are regularly excluded from the conversation. This discussion has been taken up most recently in the “Kampala Initiative,” a civil society initiative aiming to “advance cooperation and solidarity for health equity within and beyond aid” [28]. (Several members of our working group are engaged in this.)

### Bottom-up networks

Beyond the mainstream discourse on “community and civil society engagement” expressed in documents of the WHO and of international health initiatives, including the recently launched “Global Action Plan on healthy lives and well-being for all” (2019) [29], bottom-up networks and groups are working to improve accountability and promote social action and citizen engagement in health policy planning. Below we feature the networks and organizations represented in our working group: the People’s Health Movement, the Community of Practitioners on Accountability and Social Action in Health, and the World Federation of Public Health Associations. Collectively, these networks address issues of accountability, social action, and citizen engagement in health policy planning. Representatives of our working group also brought into the discussion rich experiences of homegrown efforts to strengthen people’s participation in health planning and policy processes.

The People’s Health Movement formed in 2000 when concerned individuals from diverse backgrounds in health, civil society, and academia gathered to create the first People’s Health Assembly [30]. From the Assembly emerged the People’s Charter for Health, a “statement of the shared vision, goals, principles and calls for action that unite all members of the People’s Health Movement coalition” [31]. This movement serves as a global network with the presence in about 70 countries, working to advance the Alma-Ata goal of Health for All through comprehensive, publicly supported PHC and addressing underlying determinants of health–social, environmental, political, and economic. The Community of Practitioners on Accountability and Social Action in Health (COPASAH) is a global group of practitioners who came together in 2011 to promote knowledge and capacity of community-oriented organizations and activists in the health sector who share an interest in accountability and social action. Their collective goal is “to make health systems responsive, equitable, and people-centered” [32].

The World Federation of Public Health Associations (WFPHA), founded in 1967, is one of the oldest global networking groups, representing about five million public health professionals worldwide and working with the WHO and others in “promoting global public health” [33]. In collaboration with the WHO, the WFPHA developed a Global Charter for the Public’s Health in 2015–2016, built on a long tradition of public health thinking, from the Declaration of Alma-Ata (1978) through to the Ottawa Charter (1986) and the Commission on the Social Determinants of Health (2005) [33].

EQUINET, the Regional Network on Equity in Health in East and Southern Africa, is “a network of professionals, civil society members, policy makers, state officials, and others within the region who have come together as an equity catalyst, to promote and realize shared values of equity and social justice in health” [34]. In Zimbabwe, the Community Working Group on Health (CWGH), formed in 1998, functions as a network of community-based organizations focusing on public health issues, such as user fees, unavailability of drugs in clinics, transport difficulties for people living in rural areas, and other real-life issues faced by people that reduces their access to healthcare [35]. Similarly, in Uganda, the Human Rights Research Documentation Center (HURIC) has functioned since 2012 as an “indigenous, non-profit, research documentation and advocacy organization,” promoting human rights research, implementation, and reform [36].

### People’s participation in the field of health research

Power imbalances in research collaborations between researchers in high-income countries and study subjects in low-income ones are well documented in the literature, yet power imbalances persist. To be most effective, health research should consistently address issues of benefits, representation, legitimacy, and accountability, building on generations of lessons learned “from the field”– and then go one step further. If health research is to move from an approach of “learning from the field” to jointly driving change, then proactively identifying—and correcting—power imbalances between “researchers” and “researched” is crucial. If people (individuals and groups) effectively claim their rights to participate in all stages of research processes whose outcomes affect their own health (both in biomedical and social science public health research), this might even lead to overcoming an artificial dichotomy between researchers and those ‘researched.’

Despite ethical standards for the conduct of health research, insufficient consideration has been given to the larger, macro-level ethical implications of health research.

- Is there a minimum owed to individual study participants and their communities when outside researchers conduct research in collaboration with local researchers?
- Is there a minimum set of ethical obligations and benefits owed by outside researchers to study subjects or communities under study?

Lairumbi et al. reviewed ethics guidelines internationally and found broad agreement on the sharing of benefits as a standard practice of global health research partnerships. International guidelines, however, fail to indicate a consensus on any standard or on specific ethical obligations to ensure the social value of the research, which, for Lairumbi et al., “at a minimum refers to efforts aimed at ensuring global health research contributes to improvements in human health, through for instance the generation and application of generalisable knowledge” [37]. They reference Emanuel et al. who set out benchmarks for ethical research [38]. There are straightforward ways of bringing the research back to the ‘researched.’ The World Association of Medical Editors (WAME) advocates for abstracts to be translated and published in the indigenous or local language of the study area [39].

As a step toward basing collaborative partnerships based on trust, it may be important, particularly for multi-year studies, to take a short- and long-term view. Establishing trust with people and communities takes time, beyond short-term cycles. Too often public health researchers and professionals lack understanding and investment in developing methods to provide accurate short-term and near-term predictions in public health. Yet, a science-based public health strategy requires us all to recognize and assess the consequences of any changes in public health. An ecological study from Alaska identified a key factor in its success as “reconceptualization of intervention research time frames beyond the typical three- to five-year grant funding cycle, to an enhanced understanding of how change in complex systems occurs over a time span of decades, not years” [40]. In the Netherlands, Verschuuren et al. noted how ‘foresight analysis,’ used less commonly in public health than in other fields, holds potential as a tool for working with communities to help anticipate and understand long-term needs and changes in their communities [41]. As described by Verschuuren, foresight studies combine qualitative and quantitative methods to project public health trends and policies decades into the future with the aim of developing “options” scenarios to cope with future public health challenges [41].

In our experience, participatory action research (PAR) is the most convincing methodology for changing patterns and closing the gap between researchers and those ‘researched.’ PAR arose in part, as described by Baum, through Freire’s work in the 1970s in low-income settings, where he worked with communities to question and analyze their oppression with the aim of bringing about social change [42]. Writing more than 20 years ago, Israel and colleagues identified “key principles of community-based research” that engage people being researched, including study participants, as active and full participants in knowledge creation [43].

The progress in overcoming power imbalances in health research remains varied. Full and unfettered participation of affected groups persists; box-ticking “community involvement” as “tokenization” continues. A systematic review of community participation in health systems research in LMICs from 2000 to 2012 suggests that "community participation in health systems interventions” remains uneven, “with few being truly community directed” [44]. In a nine-part series on PAR from 2019, Kjellstrom and Mitchel emphasize trust as a key element for building strong collaborative working relationships between researchers and study participants [45]. For them, research too often lacks “reflection” and “reflexivity.” They define the latter as a “more ambitious and challenging process of thinking about our own way of thinking, assumptions, and underlying patterns of values and world views” [45].

## Recommendations

We offer five concrete recommendations for public health researchers and practitioners to facilitate people’s participation in health research and policymaking. Acting on these recommendations can begin to address inadequacies and inequities that remain. Research and science should drive people’s active participation in ‘owning’ their health and their health systems.

1. Public health professionals should avoid references to “the field” as the term distances the people whose concerns should be at the center. Whenever possible, people should use names of actual people, places, and organizations in lieu of generalizations that can seem to dismiss needs, challenges, and realities of actual people, individuals, and groups, and of their life experiences.
2. Map comprehensively and analyze stakeholders’ interests to ensure all avenues of engagement are explored and pursued among affected populations;
3. Involve communities in defining research questions and disseminate research findings to the study populations, formally documenting this step;
4. Publish study findings in local languages of the study population and always acknowledge the contribution of communities;
5. Engage with local organizations that conduct health research to advocate for equity-affirming practices in public health through the use of inclusive and transparent language. Use plain language easily understood by affected people facilitates their participation, empowerment, and ultimately their ownership of study or policy results.

## Conclusion

We provide an alternative perspective on the term “in the field” using an appreciative inquiry approach to raise fundamental questions about power, rights, and knowledge ownership in global health. Far from being an optional ‘extra,’ we believe that people must be the first point of reference in analyzing barriers to access to health care, in the design of new health products or strategies, and in piloting, implementing, and monitoring health programs and health research. Many initiatives to improve people’s participation in health policymaking and research, such as those profiled above, and more broadly, the growing attention given to people’s involvement in health, health policy and planning, health practice and services, and research about all of these, illustrate a growing recognition that people must lead in analyzing and overcoming power imbalances in pursuit of more equitable health outcomes. Creating responsive public institutions that provide people with basic services is fundamental to the contract between governments and people. The next step beyond people ‘receiving’ the right services is people’s active participation in prioritizing, planning, and managing all elements of their own health systems (including health research, advocacy, and funding) and the equitable (or not) outcomes that those systems produce. We advocate greater awareness of language and its uses, as a foundational element of behaviors that spring from it, and of minimum standards to establish a common language and behavior for governments, local populations, donors, implementors, and policy makers to promote health equity. While some progress has been made in the last two decades in improving the quality of people’s engagement in health policymaking and health research, including examples above, much remains to be done to move from an approach that seeks to “learn from the field” to one that proactively and “jointly drives change” in the pursuit of more equitable health outcomes.
